# Evaluation of the Influence of Sulfur-Fumigated *Paeoniae Radix Alba* on the Quality of Si Wu Tang by Chromatographic and Chemometric Analysis

**DOI:** 10.1155/2016/8358609

**Published:** 2016-03-10

**Authors:** Ke Pei, Hao Cai, Yu Duan, Feng-Xian Qiao, Si-Cong Tu, Xiao Liu, Xiao-Li Wang, Xiao-Qing Song, Kai-Lei Fan, Bao-Chang Cai

**Affiliations:** ^1^School of Pharmacy, Nanjing University of Chinese Medicine, Nanjing 210023, China; ^2^Engineering Center of State Ministry of Education for Standardization of Chinese Medicine Processing, Nanjing University of Chinese Medicine, Nanjing 210023, China; ^3^Faculty of Medicine, University of New South Wales, Sydney, NSW 2031, Australia

## Abstract

An accurate and reliable method of high-performance liquid chromatographic fingerprint combining with multi-ingredient determination was developed and validated to evaluate the influence of sulfur-fumigated* Paeoniae Radix Alba* on the quality and chemical constituents of Si Wu Tang. Multivariate data analysis including hierarchical cluster analysis and principal component analysis, which integrated with high-performance liquid chromatographic fingerprint and multi-ingredient determination, was employed to evaluate Si Wu Tang in a more objective and scientific way. Interestingly, in this paper, a total of 37 and 36 peaks were marked as common peaks in ten batches of Si Wu Tang containing sun-dried* Paeoniae Radix Alba* and ten batches of Si Wu Tang containing sulfur-fumigated* Paeoniae Radix Alba*, respectively, which indicated the changed fingerprint profile of Si Wu Tang when containing sulfur-fumigated herb. Furthermore, the results of simultaneous determination for multiple ingredients showed that the contents of albiflorin and paeoniflorin decreased significantly (*P* < 0.01) and the contents of gallic acid and* Z*-ligustilide decreased to some extent at the same time when Si Wu Tang contained sulfur-fumigated* Paeoniae Radix Alba*. Therefore, sulfur-fumigation processing may have great influence on the quality of Chinese herbal prescription.

## 1. Introduction

In the near decades, sulfur-fumigation has been employed in postharvest handling of some medicinal herbs to keep moisture, preserve color and freshness, and prevent against insects and mildew [1]. However, sulfur-fumigation was recently reported to cause chemical transformation of original bioactive components, alter bioactivities and pharmacokinetics of typical constituents, or even generate toxicities in medicinal herbs or their extracts [2–6]. Whether therapeutic effects of traditional Chinese medicinal formulae are affected while they contain sulfur-fumigated medicinal herbs has never been reported and proved, and it is a very important issue not only for the efficacy but also for the safety of the formulae in clinical application.

Si Wu Tang (SWT), which comprises four herbs,* Rehmanniae Radix Praeparata*,* Angelicae Sinensis Radix*,* Chuanxiong Rhizoma*, and* Paeoniae Radix Alba*, is a classic formula of traditional Chinese medicine (TCM) widely used for the treatments of women's diseases such as relief of menstrual discomfort, climacteric syndrome, dysmenorrhea, and other estrogen-related diseases [7]. Some recent investigations have proved that sulfur-fumigation could cause chemical transformation of* Paeoniae Radix Alba* (an important medicinal herb in SWT) [8, 9]. However, whether sulfur-fumigated* Paeoniae Radix Alba* could influence the quality and chemical constituents of SWT has not been reported. It is widely accepted that the synergistic action of multiple constituents is responsible for the therapeutic effect of TCM while the quality of TCM is mainly controlled by the following two strategies: one is fingerprinting, which has been widely used as an efficient technique for the quality control of complex analytes, especially for TCM [10–12], and the other is multi-ingredient determination, which is usually used for assessment of quality of Chinese medicine [13]. Multivariate data analysis [14–17], such as hierarchical cluster analysis and principal component analysis, is often applied in combination with fingerprinting and multi-ingredient determination to reveal the quality of TCM.

Aimed at knowing the influence of sulfur-fumigated* Paeoniae Radix Alba* on chemical constituents of SWT, two kinds of SWT samples were investigated: one (SFP SWT) comprised three sun-dried medicinal herbs (*Rehmanniae Radix Praeparata*,* Angelicae Sinensis Radix*, and* Chuanxiong Rhizoma*) and one sulfur-fumigated medicinal herb (*Paeoniae Radix Alba*), while the other (SDP SWT) comprised four sun-dried medicinal herbs (*Rehmanniae Radix Praeparata*,* Angelicae Sinensis Radix*,* Chuanxiong Rhizoma*, and* Paeoniae Radix Alba*). Finally, a method of chemical fingerprinting analysis combining with multi-ingredient determination was developed to reveal and evaluate the influence of sulfur-fumigated* Paeoniae Radix Alba* on the quality and chemical constituents of SWT.

## 2. Experimental

### 2.1. Samples, Chemicals, and Reagents

A total of twenty batches of* Paeoniae Radix Alba* were purchased and collected from different drugstores, companies, and GAP bases (Table 1). Among them, ten batches (from number 1 to number 10) were proved to be sulfur-fumigated samples and ten batches (from number 11 to number 20) were proved to be sun-dried ones by the method for determination of sulfur dioxide residue included in Chinese Pharmacopoeia [18]. One batch of* Chuanxiong Rhizoma*, one batch of* Angelicae Sinensis Radix*, and one batch of* Rehmanniae Radix Praeparata* were collected from Sichuan, Gansu, and Henan province, China, respectively, and proved to be sun-dried samples by the above method for determination of sulfur dioxide residue. These herbal samples were authenticated by Professor Hao Cai. The voucher specimens were stored in the Herbarium of School of Pharmacy, Nanjing University of Chinese Medicine (Nanjing, PR China).

The reference standards of gallic acid (M-017-120911), 5-hydroxymethylfurfural (Q-042-131226), catechin (E-011-121116), albiflorin (S-011-121206), paeoniflorin (S-010-130429), ferulic acid (A-002-121011), acteoside (M-011-130115), senkyunolide I (Y-083-130113), senkyunolide A (Y-083-130113), and* Z*-ligustilide (G-010-130428) with purities of 98% or higher were all purchased from the Chengdu Herbpurify Co., Ltd. (Chengdu, China). Methanol (HPLC grade) and formic acid (HPLC grade) were purchased from E. Merck (Darmstadt, Germany). Purified water was acquired from a Milli-Q system (Millipore, Bedford, MA, USA).

### 2.2. Instrumentation and High-Performance Liquid Chromatographic Conditions

The fingerprinting analysis was performed on 920-LC HPLC system (Varian, USA) equipped with Prostar 240 quatpump, Prostar 410 autosampler, Prostar 335 DAD, and Galaxie Chemstation, and the quantitative analysis of ten bioactive constituents was performed on e2695 HPLC system (Waters, USA) equipped with 2998 photodiode array detector and Empower 2 Chemstation. The chromatographic separation was carried out on a Hypersil ODS C_18_ column (250 mm × 4.6 mm, 5 *µ*m) under column temperature of 30°C at a flow rate of 0.8 mL/min. The detection wavelength was set at 280 nm and the injection volume was 20 *μ*L. The mobile phase was composed of 0.3% aqueous formic acid (A) and methanol (B). The gradient elution program for fingerprinting analysis was as follows: 0–5 min, 2% A, 5–18 min, 2%–20% A, 18–30 min, 20% A, 30–80 min, 20%–70% A, and 80–90 min, 70%–100% A. However, for multi-ingredient analysis, the gradient elution program was as follows: 0–2 min, 2% A, 2–15 min, 2%–20% A, 15–25 min, 20% A, 25–42 min, 20%–100% A, and 42–45 min, 100% A.

### 2.3. Preparation of Standard Solutions

**Figure 1:**
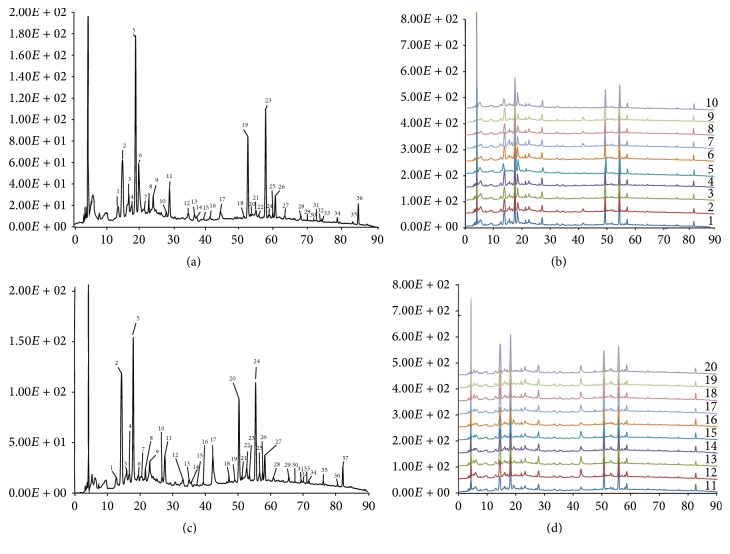
Typical LC profiles of 36 common peaks (a) and comparison of chromatographic fingerprints of SFP SWT (b), and typical LC profiles of 37 common peaks (c) and comparison of chromatographic fingerprints of SDP SWT (d). Gallic acid (14.12 min), 5-hydroxymethylfurfural (17.93 min), cianidanol (27.79 min), albiflorin (36.83 min), paeoniflorin (42.57 min), ferulic acid (50.56 min), verbascoside (53.72 min), senkyunolide I (55.72 min), senkyunolide A (76.55 min), and* Z*-ligustilide (82.63 min).

Each accurately weighed standard was dissolved in methanol, respectively, and then mixed and diluted to get a stock standard solution with concentrations of 182.00 *µ*g/mL for gallic acid, 64.40 *µ*g/mL for 5-hydroxymethylfurfural, 239.00 *µ*g/mL for cianidanol, 789.00 *µ*g/mL for albiflorin, 357.00 *µ*g/mL for paeoniflorin, 17.00 *µ*g/mL for ferulic acid, 61.00 *µ*g/mL for verbascoside, 78.00 *µ*g/mL for senkyunolide I, 158.50 *µ*g/mL for senkyunolide A, and 123.20 *µ*g/mL for* Z*-ligustilide. The working standard solutions were prepared daily by diluting the primary stock standard solution with methanol to get different concentrations for calibration curves. The RP-HPLC chromatogram of mixed standards is shown in Figure 1. All the standard solutions were stored in refrigerator at 4°C before analysis.

### 2.4. Preparation of SDP SWT and SFP SWT Sample Solutions

For preparation of twenty batches of SWT, four medicinal herbs used in composition of SWT were mixed as follows: twenty batches (from batch number 1 to number 20) of* Paeoniae Radix Alba* were mixed, respectively, with the other medicinal herbs (*Chuanxiong Rhizoma*,* Angelicae Sinensis Radix*, and* Rehmanniae Radix Praeparata*) coming from the same batch.

The preparation of SDP SWT was as follows: sun-dried* Paeoniae Radix Alba* (10 g), sun-dried* Chuanxiong Rhizoma* (6 g), sun-dried* Angelicae Sinensis Radix* (10 g), and sun-dried* Rehmanniae Radix Praeparata* (15 g) were weighed accurately according to the classic percentage of clinical dose and mixed well. The mixed sun-dried medicinal herbs were soaked in distilled water (10 : 1, v/w) for 30 min and then boiled for 1 h and extracted twice (the preparation method followed the ancient method and was also same with the clinical preparation). Finally, the extracts were filtered, combined, and concentrated to 300 mL under vacuum by using a rotary evaporator. 4 mL of the concentrated extract was precisely measured, then transferred into a dark brown calibrated flask, and extracted with 6 mL of methanol in an ultrasonic bath for 30 min. Additional 60% (v/v) methanol was added to make up the loss after standing to the room temperature. The obtained solution was filtered through a 0.45 *μ*m membrane filter before injection into the HPLC system for analysis.

Similarly, the preparation of SFP SWT was in the same way with that of SDP SWT by using sulfur-fumigated* Paeoniae Radix Alba*, sun-dried* Chuanxiong Rhizoma*, sun-dried* Angelicae Sinensis Radix*, and sun-dried* Rehmanniae Radix Praeparata*.

### 2.5. Data Analysis

The chromatographic profiles of all SWT samples were performed by using professional software entitled “Similarity Evaluation System for Chromatographic Fingerprint of Traditional Chinese Medicine (Version 2004A).” The hierarchical cluster analysis (HCA) and the principal component analysis (PCA) of SWT samples were performed by using SPSS software (SPSS 16.0 for Windows Vista*™*, SPSS Inc., Chicago, IL, USA).

## 3. Results

### 3.1. Chromatographic Fingerprinting Analysis

#### 3.1.1. Optimization of Extraction Methods and Chromatographic Conditions

In the experiment, different soaking time (20 min, 30 min, and 40 min), different extraction times (1, 2, and 3), and different methanol compositions (60%, 70%, and 80%, v/v) were tried to obtain the optimal chromatograms with good resolution and abundant peaks. Finally, 30 min of soaking time, 2 of extraction times, and 60% of methanol were selected to give the desired chromatograms.

Formic acid was added into the mobile phase to prevent the tailing of chromatographic peaks and improve the separation of compounds. The effects of different concentrations of aqueous formic acid (0.1%, 0.2%, and 0.3%, v/v) were investigated, and it was found that 0.3% aqueous formic acid (phase A) could get better separation and peak shapes. Furthermore, other chromatographic variables, including column temperatures (25°C, 30°C, and 35°C), flow rates (0.8 mL/min and 1.0 mL/min), and detection wavelengths (230 nm, 254 nm, and 280 nm), were also optimized. Eventually, the optimal chromatographic separation was achieved at a column temperature of 30°C with a flow rate of 0.8 mL/min and a detection wavelength of 280 nm.

#### 3.1.2. Establishment of Common Patterns of SDP SWT and SFP SWT and Calculation of RRT and RPA

A software used in the similarity analysis of chromatographic and spectral patterns based on chemometrics and recommended by SFDA, entitled “Similarity Evaluation System for Chromatographic Fingerprint of Traditional Chinese Medicine (Version 2004A),” was employed to establish the chromatographic common patterns of SDP SWT and SFP SWT. The similarity evaluation system for chromatographic fingerprint of TCM could reflect the similarity of the distribution ratio of the chemical composition accurately, rather than as a function of the quantitative evaluation.

The common patterns of SDP SWT and SFP SWT were shown in Figure 1. Ten compounds were identified by comparing their retention behaviors and UV characteristics with standards. Among them, ferulic acid was selected as reference for RRT and RPA calculation since its peak was symmetrical and detectable in all tested samples. The calculation formulas of RRT and RPA were RRT = RT_peak_/RT_reference_ and RPA = PA_peak_/PA_reference_, respectively. The purpose to calculate RRT and RPA was to make the various absolute values become relatively stable, which could semiquantitatively reflect the constituents displayed in the chromatographic profiles of the samples.

#### 3.1.3. Method Validation

The injection precision was assessed by replicated injection of the same sample six times in one day. The relative standard deviations (RSD) of RRT and RPA were lower than 2.3% and 3.1%, respectively. The repeatability was evaluated by assessing six independently prepared samples from the same batch of the SDP SWT. The RSD of RRT and RPA were lower than 2.7% and 6.2%, respectively. The sample stability was assessed by injecting same sample in 0, 2, 4, 6, 8, 10, 12, and 24 h, respectively. The RSD of RRT and RPA were lower than 2.1% and 7.4%, respectively.

#### 3.1.4. Analysis of Chromatographic Fingerprints of SDP SWT and SFP SWT

The chromatographic fingerprints showed abundant diversity of chemical constituents between SDP SWT and SFP SWT from different populations (Figure 1). In this paper, a LC chemical fingerprinting method was utilized and developed for distinguishing SDP SWT and SFP SWT for the first time. Under the optimized conditions of extraction and chromatographic separation, well-separated and reproducible chromatograms were achieved. A total of 36 and 37 peaks were marked as common peaks in the chromatograms of ten batches of SFP SWT and ten batches of SDP SWT, respectively. Ten peaks were identified as gallic acid (14.12 min), 5-hydroxymethylfurfural (17.93 min), cianidanol (27.79 min), albiflorin (36.83 min), paeoniflorin (42.57 min), ferulic acid (50.56 min), verbascoside (53.72 min), senkyunolide I (55.72 min), senkyunolide A (76.55 min), and* Z*-ligustilide (82.63 min) by comparing to the standards. Compared to the common peak pattern of SDP SWT, the areas of peak number 6 (18.83 min) and peak number 17 (42.57 min) in the common peak pattern of SFP SWT were increased and decreased significantly at the same time, respectively. Meanwhile, peak number 18 (47.51 min) in the common peak pattern of SDP SWT was disappeared in the common peak pattern of SFP SWT. Aimed at evaluating the differences more accurately and objectively, the multi-ingredient quantitative analysis was carried out subsequently.

#### 3.1.5. Hierarchical Cluster Analysis

HCA is a multivariate analysis technique, which can provide a visual representation of complex data. Thus, it can be used to classify samples according to their own data characters. Besides, HCA can apply for fingerprint analysis, because it is a nonparametric data interpretation method and simple to use. SPSS is a kind of software that can do hierarchical cluster analysis conveniently [19].

**Figure 2:**
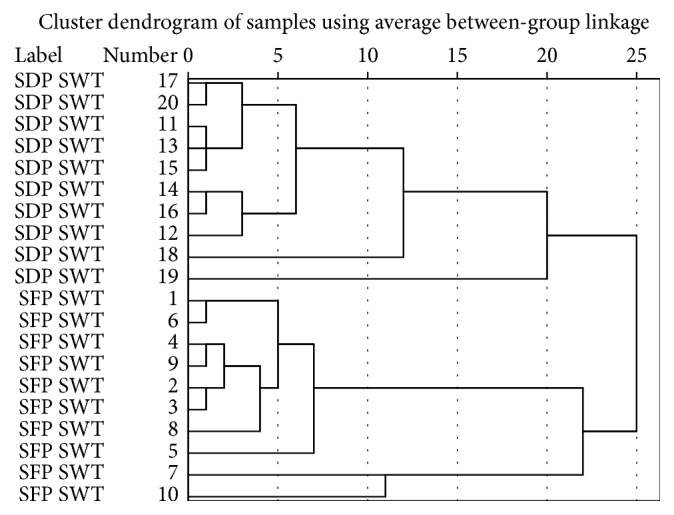
Hierarchical clustering dendrogram of twenty batches of SWT (1–10, SFP SWT; 11–20, SDP SWT).

In order to discriminate the differences of these samples, the areas of common peaks of SWT samples (1–20) were calculated, imported into the software of SPSS, and standardized for clustering analysis. Then the groups were set as variables, which were objects of cluster. The between-group linkage was used as cluster method, in which the interval was measured by squared Euclidean distance. After calculating by SPSS, the results of hierarchical cluster analysis of 20 batches of SWT samples were obtained. The results (Figure 2) showed that the samples were divided into two groups at squared Euclidean distance between 15 and 20. All SFP SWT (1–10) were clustered into one group while all SDP SWT (11–20) were clustered into the other group. It indicated that sulfur-fumigated* Paeoniae Radix Alba* could change the fingerprinting characteristic of SWT, which closely links to the chemical composition of SWT.

### 3.2. Quantitative Analysis

#### 3.2.1. Method Validation

*(1) Calibration Curves, Limits of Detection, and Limits of Quantification*. The calibration curves were plotted with a series of concentrations of standard solutions. As shown in Table 2, all analytes showed good linearity (*r* ^2^ > 0.9990) in a relatively wide concentration ranges. The limits of detection (LOD) and the limits of quantification (LOQ) were determined by injecting a series of dilute solutions with known concentrations besides considering the concentrations giving a signal-to-noise ratio of 3 and 10, respectively. The results (Table 2) showed that LOD and LOQ of ten marker compounds were all within the ranges of 0.02–3.71 *μ*g/mL and 0.06–12.25 *μ*g/mL, respectively, revealing a high sensitivity under the established chromatographic conditions. 

*(2) Precision, Repeatability, Stability, and Recovery*. The precision of the method was validated by determination of the intra- and interday variability. The intraday precision was determined by injection of the same standard solution six consecutive times in the same day, while the interday values were carried out by duplicating the experiments on three consecutive days. The RSD values were calculated and Table 2 showed that the intra- and interday RSD values of ten compounds were all less than 2.82%. To evaluate the repeatability of the method, six independently prepared solutions from the same sample were analyzed. The RSD values were all among 1.77%–3.03%. The stability of sample solution was tested at room temperature. The sample solution was analyzed at 0, 2, 4, 6, 8, 10, 12, and 24 h, the RSD was shown to be less than 2.84%, indicating sample solutions were stable within 24 h. The recovery tests were carried out to investigate the accuracy of the method by spiking known amounts of the mixed standard solutions to SWT in which the content of ten analytes had been quantified. The resultant samples were then extracted and analyzed with the above described method for preparation of sample solutions. Six replicates were performed for the determination. The recoveries of ten compounds were ranged within 98.45%–102.75% with RSD ≤ 2.73%.

#### 3.2.2. Sample Analysis

**Figure 3:**
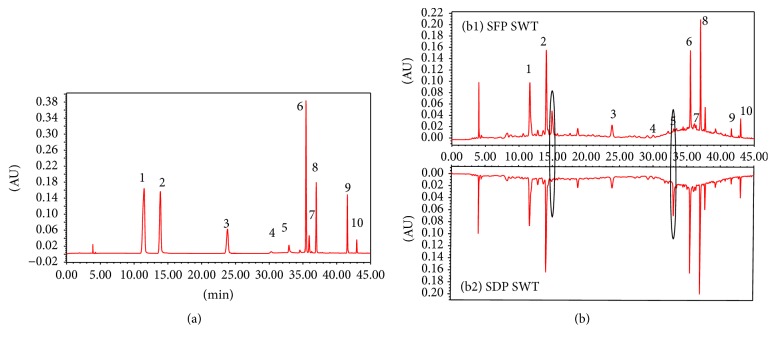
Typical HPLC chromatograms of mixed standard solution (a) and SWT sample solutions (b) ((b1) SFP SWT; (b2) SDP SWT). (1) Gallic acid, (2) 5-hydroxymethylfurfural, (3) cianidanol, (4) albiflorin, (5) paeoniflorin, (6) ferulic acid, (7) verbascoside, (8) senkyunolide I, (9) senkyunolide A, and (10)* Z*-ligustilide.

The developed quantitative analysis method was subsequently applied to simultaneous quantitative analysis of ten components in twenty batches of SWT (from number 1 to number 10, SFP SWT; from number 11 to number 20, SDP SWT). The results demonstrated a successful application of HPLC-DAD assay for quantification of ten compounds in different samples. All ten compounds were eluted and separated within 50 min. Representative HPLC-DAD chromatograms of mixed standard solution and SWT sample solutions were shown in Figure 3. The contents of ten compounds in twenty batches of SWT were summarized in Table 3.

**Figure 4:**
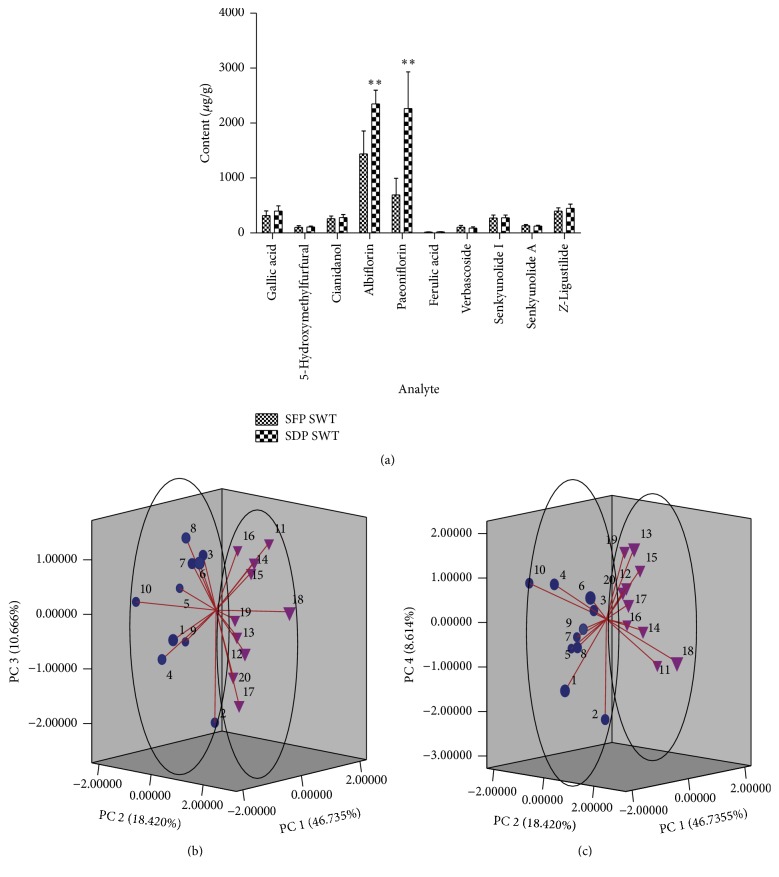
Column histogram statistics of content comparison (^*∗∗*^ *P* < 0.01) (a), PC 1-PC 2-PC 3 score plot of PCA for twenty batches of SWT (b), and PC 1-PC 2-PC 4 score plot of PCA for twenty batches of SWT (c) (1–10, SFP SWT; 11–20, SDP SWT).

As seen from Table 3 and Figure 4, compared to SDP SWT, the contents of albiflorin and paeoniflorin in SFP SWT decreased significantly (*P* < 0.01), which indicated that albiflorin and paeoniflorin were more sensitive to the processing of sulfur-fumigation. And also the contents of gallic acid and* Z*-ligustilide in SFP SWT decreased to some extent at the same time. However, albiflorin, paeoniflorin, and gallic acid have been proved to be the main bioactive ingredients in* Radix Paeoniae Alba *and* Z*-ligustilide is the main bioactive ingredient in* Chuanxiong Rhizoma* and* Angelicae Sinensis Radix*; therefore, the clinical efficacy of SWT containing sulfur-fumigated* Radix Paeoniae Alba* might be affected.

#### 3.2.3. Principal Component Analysis

PCA is a well-known, unsupervised, bilinear, pattern recognition method [20]. The initial data matrix is converted into a set of orthogonal variables called principal components (PCs), and the model consists of linear equations, each of which has as many terms as the original variables. PC 1 accounts for most of the data variance, PC 2 accounts for the next largest amount, and so on until all data variance is accounted for. Each PC is characterized by its loading value and every sample-object has a score value on PC [21].

As seen from Figure 4, sulfur-fumigated* Paeoniae Radix Alba* could affect the contents of compounds in SWT. In order to evaluate the discrimination ability of these components, PCA was carried out by using the calculated concentrations of ten ingredients of SWT as input data. On the basis of eigenvalues >0.7, the first four principal components PC 1 (46.735%), PC 2 (18.420%), PC 3 (10.666%), and PC 4 (8.614%) were extracted, and the accumulative contribution rate of these four factors to the total variation accounted for over 84%, maintaining most of information of ten characters. The score plots of PC 1, PC 2, and PC 3 and PC 1, PC 2, and PC 4 (Figure 4) showed the clear differentiation of SFP SWT and SDP SWT. These results were corresponding with hierarchical cluster analysis. The results of hierarchical cluster analysis and principal component analysis could be validated with each other and provided more references for the quality evaluation of SFP SWT and SDP SWT.

## 4. Discussion

The different chemical patterns between SDP SWT and SFP SWT shown in chromatographic fingerprinting analysis indicated the changed ingredients in SWT when containing sulfur-fumigated* Paeoniae Radix Alba*. Moreover, the cluster analysis, which has been applied to clearly separate SFP SWT and SDP SWT successfully, could be combined to chromatographic fingerprinting analysis to identify sulfur-fumigation of other traditional Chinese medicines. Subsequently, multi-ingredient quantitative analysis was carried out for evaluating the differences of SFP SWT and SDP SWT, found in chromatographic fingerprinting analysis, more accurately and objectively, and the results indicated that the contents of albiflorin, paeoniflorin, and gallic acid, three bioactive ingredients in* Paeoniae Radix Alba*, were all decreased at the same time in SFP SWT compared to SDP SWT. Besides, the content* Z*-ligustilide, which has been reported as the main bioactive compound in* Angelicae Sinensis Radix* and* Chuanxiong Rhizoma, *also decreased unexpectedly in SFP SWT, which indicated that the sulfur-fumigated* Paeoniae Radix Alba *may also affect the compounds of other medicinal herbs in SWT. Furthermore, the clear discrimination of SFP SWT and SDP SWT in PCA analysis indicated that all above compounds with changed contents contribute to the differentiation of SFP SWT and SDP SWT when the calculated concentrations of ten ingredients in twenty batches of SWT were set as matrix.

## 5. Conclusions

In this paper, chromatographic fingerprints were acquired to evaluate the quality of SFP SWT and SDP SWT qualitatively, while the method of simultaneous determination of ten compounds which were identified by comparing their retention behaviors and UV characteristics with the reference compounds could evaluate the quality of SFP SWT and SDP SWT quantificationally. Additionally, multivariate statistical methods including clustering analysis and principal component analysis were employed to combine with the fingerprinting analysis and multi-ingredient quantitative analysis to distinguish SFP SWT and SDP SWT in a more objective and scientific way. Eventually, the results indicated that sulfur-fumigated medicinal herb could actually influence the inherent chemical feature of its corresponding formula. As the chemical feature of the formula was directly related to its pharmacological activities and clinical safety, the influence of sulfur-fumigation on the formula could not be ignored and should be paid high attention.

## Figures and Tables

**Table 1:** Sources and processing methods of twenty batches of *Paeoniae Radix Alba*.

Sample number	Source	Processing method
1	Nanjing Haichang Chinese Medicine Group Corporation, Nanjing (121101)	Sulfur-fumigated
2	Nanjing Haichang Chinese Medicine Group Corporation, Nanjing (121107)	Sulfur-fumigated
3	Nanjing Haichang Chinese Medicine Group Corporation, Nanjing (121113)	Sulfur-fumigated
4	Nanjing Haichang Chinese Medicine Group Corporation, Nanjing (121121)	Sulfur-fumigated
5	Nanjing Haichang Chinese Medicine Group Corporation, Nanjing (120123)	Sulfur-fumigated
6	Nanjing Haichang Chinese Medicine Group Corporation, Nanjing (120207)	Sulfur-fumigated
7	Nanjing Haichang Chinese Medicine Group Corporation, Nanjing (120213)	Sulfur-fumigated
8	Yida Drugstore, Nanjing (120505)	Sulfur-fumigated
9	Yifeng Drugstore, Nanjing (120501)	Sulfur-fumigated
10	Yifeng Drugstore, Nanjing (111001)	Sulfur-fumigated
11	Sijichangqing Drugstore, Sichuan Province	Sun-dried
12	Xinxin Drugstore, Anhui Province	Sun-dried
13	Chinese Herbal Pieces Company, Bozhou (20120324)	Sun-dried
14	Youyoubencao Drugstore, Sichuan Province	Sun-dried
15	GAP Base, Shanxi Province	Sun-dried
16	Chinese Herbal Pieces Company, Bozhou (120326)	Sun-dried
17	Chinese Herbal Pieces Company, Bozhou (120328)	Sun-dried
18	Youyoubencao Drugstore, Sichuan Province	Sun-dried
19	Hangzhou, Zhejiang Province	Sun-dried
20	Hangzhou, Zhejiang Province	Sun-dried

**Table 2:** Linear regression data, LOD, LOQ, precisions, repeatabilities, and recoveries of ten marker compounds in SWT (*n* = 6).

Analyte	Regression equation	*r* ^2^	Linear range (*µ*g/mL)	LOD (*µ*g/mL)	LOQ (*µ*g/mL)	Precision, RSD (%)		Repeatability RSD (%)	Recovery	
						Intraday	Interday		Average recovery (%)	RSD (%)
Gallic acid	*y* = 32653*x* − 80572	0.9990	11.30–182.00	1.70	5.65	1.10%	1.9%	2.16%	99.67	1.31
5-Hydroxymethylfurfural	*y* = 139478*x* − 211161	0.9994	4.00–64.40	0.02	0.06	0.26%	0.43%	1.77%	101.58	1.25
Cianidanol	*y* = 12187*x* − 49640	0.9990	14.00–239.00	0.53	1.75	0.49%	0.59%	3.03%	101.37	2.17
Albiflorin	*y* = 303.26*x* − 8112.8	0.9988	49.00–789.00	3.71	12.25	1.44%	2.12%	2.72%	98.45	2.33
Paeoniflorin	*y* = 1733*x* − 22795	0.9990	22.30–357.00	1.69	5.57	2.00%	2.31%	2.92%	102.75	1.78
Ferulic acid	*y* = 53532*x* − 17483	0.9991	1.06–17.00	0.02	0.07	0.38%	1.05%	2.86%	99.63	1.15
Verbascoside	*y* = 13848*x* − 5314.4	0.9994	3.80–61.00	0.15	0.48	2.73%	2.82%	2.87%	98.69	2.73
Senkyunolide I	*y* = 37197*x* − 44264	0.9998	4.80–78.00	0.04	0.15	0.35%	0.54%	2.97%	102.55	2.06
Senkyunolide A	*y* = 6169.6*x* − 3579.4	0.9989	9.91–158.50	0.18	0.61	0.61%	0.93%	2.61%	101.58	2.15
*Z*-Ligustilide	*y* = 3879*x* − 12478	0.9990	11.30–182.00	0.86	2.83	0.21%	0.37%	2.41%	99.67	1.79

**Table 3:** Contents (*µ*g/g) of ten marker compounds in twenty batches of SWT (1–10, SFP SWT; 11–20 SDP SWT).

Sample number	Gallic acid	5-Hydroxymethylfurfural	Cianidanol	Albiflorin	Paeoniflorin	Ferulic acid	Verbascoside	Senkyunolide I	Senkyunolide A	*Z*-Ligustilide
1	284.03	139.05	321.51	817.42	853.85	25.07	119.68	351.24	143.18	422.18
2	444.26	118.85	317.50	1467.22	843.54	23.17	104.36	345.63	91.32	387.92
3	356.84	109.28	290.29	2320.99	322.86	20.56	105.04	283.48	153.59	405.33
4	272.74	68.42	234.70	1278.80	910.13	23.15	143.30	286.17	137.06	378.08
5	267.22	71.52	217.59	1336.35	218.92	17.76	62.83	233.50	128.19	394.19
6	471.54	133.70	326.21	1884.37	612.68	27.56	168.68	321.97	168.89	491.00
7	384.16	148.58	246.65	1392.91	974.82	20.61	106.84	249.63	144.57	417.55
8	276.42	124.13	266.14	1434.81	922.51	17.84	111.14	283.45	150.77	490.81
9	215.68	78.36	241.35	1468.71	1001.86	18.29	71.53	246.73	118.83	352.67
10	233.76	91.09	173.89	1052.40	307.11	14.16	113.40	192.70	133.62	297.75
11	493.45	108.53	258.50	2713.62	1112.21	20.01	55.59	285.55	123.71	519.16
12	318.58	115.71	260.90	2063.30	2856.41	23.61	97.62	266.08	128.39	427.92
13	505.48	119.03	332.68	2280.59	2838.06	27.46	113.81	301.19	153.96	355.63
14	409.28	124.54	292.12	2366.80	1682.88	24.61	60.21	274.31	143.43	464.23
15	323.10	98.85	255.41	2850.16	2429.63	21.42	98.09	279.60	141.32	478.51
16	276.01	98.39	208.32	2423.69	1381.20	19.22	80.84	215.25	115.53	530.82
17	355.36	113.77	301.43	2159.95	2671.40	26.14	113.15	305.60	114.26	386.40
18	546.11	153.56	410.26	2252.70	3098.41	30.85	117.94	386.84	146.34	599.60
19	469.81	110.88	236.22	2170.68	2316.08	22.33	104.59	211.56	128.89	386.62
20	358.05	101.65	282.46	2255.02	2318.40	23.98	116.27	285.03	117.08	399.53
Mean ± SD (1–10)	320.67 ± 80.01	108.33 ± 26.48	263.53 ± 45.41	1445.40 ± 415.23^*∗∗*^	696.83 ± 305.53^*∗∗*^	20.82 ± 3.61	110.68 ± 27.68	279.45 ± 45.74	137.00 ± 19.27	403.75 ± 52.72
Mean ± SD (11–20)	405.52 ± 92.81	114.49 ± 16.22	283.83 ± 56.47	2353.65 ± 249.83	2270.47 ± 668.25	23.96 ± 3.53	95.81 ± 22.92	258.94 ± 87.48	131.29 ± 14.16	454.84 ± 77.80

^*∗∗*^ *P* < 0.01, compared to SDP SWT.

## References

[1] Liu J.-J., Liu X., Li S.-L., Cai B.-C., Cai H. (2010). Current situation in studies on traditional Chinese medicinal materials and Yinpian by sulfur-fumigated process. *Chinese Traditional and Herbal Drugs*.

[2] Hayes P. Y., Lehmann R., Penman K., Kitching W., De Voss J. J. (2005). Sodium paeoniflorin sulfonate, a process derived artefact from paeoniflorin. *Tetrahedron Letters*.

[3] Li S.-L., Song J.-Z., Choi F. F. K. (2009). Chemical profiling of Radix Paeoniae evaluated by ultra-performance liquid chromatography/photo-diode-array/quadrupole time-of-flight mass spectrometry. *Journal of Pharmaceutical and Biomedical Analysis*.

[4] Wang X.-H., Xie P.-S., Lam C. W. K., Yan Y.-Z., Yu Q.-X. (2009). Study of the destructive effect to inherent quality of *Angelicae dahuricae* radix (Baizhi) by sulfur-fumigated process using chromatographic fingerprinting analysis. *Journal of Pharmaceutical and Biomedical Analysis*.

[5] Wang Q., Liu R.-X., Guo H.-Z., Zhu Z.-N., Bi K.-S., Guo D.-A. (2006). Study on influence of processing methods on chemical constituents in Radix Paeoniae Alba. *China Journal of Chinese Materia Medica*.

[6] Cheng Y. S., Peng C., Wen F. Y., Zhang H. (2010). Pharmacokinetic comparisons of typical constituents in white peony root and sulfur fumigated white peony root after oral administration to mice. *Journal of Ethnopharmacology*.

[7] Hao Q.-X., Wang J.-F., Niu J.-Z., Zhao P.-W., Cui Y. (2008). Effects of the pharmacological serum of Siwu Decoction and its ingredient herbs on proliferation and cell cycle of breast cancer cell line MCF-7 cells. *Acta Chinese Medicine and Pharmacology*.

[8] Liu J.-J., Liu X., Cai H., Li S.-L., Cai B.-C. (2010). Further investigation on the reasons for contents of paeoniflorin in commercial Radix Paeoniae Alba of prepared Chinese crude drug lower than the standard of Chinese Pharmacopeia. *Chinese Journal of Pharmaceutical Analysis*.

[9] Zhang J. D., Cai H., Cao G., Liu X., Wen C. P., Fan Y. S. (2013). Exploring potential chemical transformation by chemical profiling approach for rapidly evaluating chemical consistency between sun-dried and sulfur-fumigated Radix Paeoniae Alba using ultraperformance liquid chromatography coupled with time-of-flight mass spectrometry. *Evidence-Based Complementary and Alternative Medicine*.

[10] Dumarey M., van Nederkassel A. M., Deconinck E., Vander Heyden Y. (2008). Exploration of linear multivariate calibration techniques to predict the total antioxidant capacity of green tea from chromatographic fingerprints. *Journal of Chromatography A*.

[11] Kang J., Zhou L., Sun J.-H., Han J., Guo D.-A. (2008). Chromatographic fingerprint analysis and characterization of furocoumarins in the roots of *Angelica dahurica* by HPLC/DAD/ESI-MS^n^ technique. *Journal of Pharmaceutical and Biomedical Analysis*.

[12] Bartolomé L., Deusto M., Etxebarria N., Navarro P., Usobiaga A., Zuloaga O. (2007). Chemical fingerprinting of petroleum biomarkers in biota samples using retention-time locking chromatography and multivariate analysis. *Journal of Chromatography A*.

[13] Wang Z.-J., Wo S.-K., Wang L. (2009). Simultaneous quantification of active components in the herbs and products of Si-Wu-Tang by high performance liquid chromatography-mass spectrometry. *Journal of Pharmaceutical and Biomedical Analysis*.

[14] Ni Y. N., Peng Y. Y., Kokot S. (2008). Fingerprinting of complex mixtures with the use of high performance liquid chromatography, inductively coupled plasma atomic emission spectroscopy and chemometrics. *Analytica Chimica Acta*.

[15] Yi L.-Z., Yuan D.-L., Liang Y.-Z., Xie P.-S., Zhao Y. (2007). Quality control and discrimination of *Pericarpium Citri Reticulatae* and *Pericarpium Citri Reticulatae Viride* based on high-performance liquid chromatographic fingerprints and multivariate statistical analysis. *Analytica Chimica Acta*.

[16] Rezzi S., Axelson D. E., Héberger K., Reniero F., Mariani C., Guillou C. (2005). Classification of olive oils using high throughput flow ^1^H NMR fingerprinting with principal component analysis, linear discriminant analysis and probabilistic neural networks. *Analytica Chimica Acta*.

[17] Chen Y., Zhu S.-B., Xie M.-Y. (2008). Quality control and original discrimination of *Ganoderma lucidum* based on high-performance liquid chromatographic fingerprints and combined chemometrics methods. *Analytica Chimica Acta*.

[18] Chinese Pharmacopoeia Commission (2010). *Chinese Pharmacopoeia*.

[19] Wu Y.-B., Zheng L.-J., Yi J., Wu J.-G., Chen T.-Q., Wu J.-Z. (2013). Quantitative and chemical fingerprint analysis for the quality evaluation of receptaculum nelumbinis by RP-HPLC coupled with hierarchical clustering analysis. *International Journal of Molecular Sciences*.

[20] Dong W.-J., Ni Y.-N., Kokot S. (2012). Quantitative analysis of two adulterants in *Cynanchum stauntonii* by near-infrared spectroscopy combined with multi-variate calibrations. *Chemical Papers*.

[21] Ding X.-X., Ni Y.-N., Kokot S. (2014). Differentiation of cultivars of *Flos Chrysanthemum* with the use of high-performance liquid chromatography fingerprints and chemometrics. *Analytical Letters*.

